# Association between platelet-to-red cell distribution width ratio and all-cause mortality in critically ill patients with non-traumatic cerebral hemorrhage: a retrospective cohort study

**DOI:** 10.3389/fneur.2024.1456884

**Published:** 2024-11-28

**Authors:** Rongrong Lu, Changcai Wu

**Affiliations:** ^1^Department of Neurosurgery, The Second Affiliated Hospital and Yuying Children's Hospital of Wenzhou Medical University, Wenzhou, Zhejiang, China; ^2^Department of Ultrasound, The First Affiliated Hospital of Wenzhou Medical University, Wenzhou, Zhejiang, China

**Keywords:** platelets to red cell distribution width ratio, non-traumatic cerebral hemorrhage, MIMIC-IV database, all-cause mortality, critically ill patients

## Abstract

**Background:**

The purpose of this study was to investigate the relationship between platelet-to-red cell distribution width ratio (PRR) and all-cause mortality in critically ill patients with non-traumatic cerebral hemorrhage (NCH).

**Methods:**

The Medical Information Mart for Intensive Care (MIMIC-IV) database was used to identify patients with NCH who needed to be admitted to intensive care unit (ICU). The outcomes of the study included both ICU and in-hospital mortality. Restricted cubic splines and Cox proportional hazards regression analysis were used to clarify the relationship between PRR and clinical outcomes in critically ill patients with NCH.

**Results:**

A total of 3,094 patients (54.0% male) were included in the study, with in-hospital mortality and ICU mortality rates of 16.5 and 11.8%, respectively. A substantial correlation was found by multivariate Cox proportional hazards analysis between increased PRR and a lower risk of in-hospital and ICU mortality. Following adjustment for confounding factors, patients with elevated PRR exhibited a significantly decreased risk of in-hospital death (HR, 0.98; 95% CI, 0.96–0.99; *p* = 0.006) and ICU death (HR, 0.98; 95% CI, 0.96–0.99; *p* = 0.027). As PRR increased, restrictive cubic splines showed a progressive decrease in the probability of all-cause mortality. Stratified analyses indicated a consistent association between PRR and both in-hospital and ICU mortality.

**Conclusion:**

Among critically ill patients with NCH, lower PRR was substantially correlated with the increased probability of all-cause mortality in both the ICU and hospital. According to this research, PRR might be a valuable indicator for identifying NCH patients at risk of all-cause mortality.

## 1 Introduction

Non-traumatic cerebral hemorrhage (NCH) is categorized into several types based on the hemorrhage location: intracerebral hemorrhage (ICH), subarachnoid hemorrhage (SAH), subdural hemorrhage, epidural hemorrhage, and others. The causes of NCH are diverse, with primary factors including hypertension, cerebral amyloid angiopathy, aneurysms, and vascular malformations (1). Among these, ICH is the second most prevalent type of stroke, comprising about 20% of all strokes, while SAH accounts for approximately 5%. These conditions are significant health concerns globally due to their severity, rapid progression, and high rates of mortality, disability (2–4). Patients with NCH face a significant threat to their lives. Despite optimal care in ICU and during hospitalization, hospital mortality rates remain high for patients with NCH (5). Epidemiological studies have revealed that hospital mortality rates for NCH can reach 20%, and are even higher in developing countries (5, 6). Given the potentially fatal nature of this illness, non-invasive, affordable diagnostic techniques are desperately needed to identify individuals at higher risk of mortality and help reduce fatalities (7).

The platelet counts and red blood cell distribution width (RDW) provide information on the hemostasis state and the heterogeneity of red blood cell volume in peripheral blood. Platelets play crucial roles in blood clotting, immune defense, and tissue repair (8, 9). RDW is a strong predictor of bloodstream infection risk and all-cause mortality in critically ill patients, and it has gained attention as an inflammatory marker (10–12). Changes in PRR may reflect the body’s inflammatory state and disease prognosis in conditions such as acute kidney injury (13), sepsis (14), and acute traumatic brain injury (15). Lower PRR levels are typically associated with more severe inflammatory responses and poor prognosis. In patients with esophageal cancer, PRR correlates with TNM stage of tumor (16). Recently, in patients with deep intracerebral hemorrhage, Lehmann et al. (17) explored the connection between PRR and 90-day mortality. However, their study was limited by a small sample size and excluded individuals with additional sources of bleeding. Consequently, the relationship between PRR and mortality in patients with severe cerebral hemorrhage was yet unclear.

The objective of this study was to examine the association between PRR and mortality in NCH patients both in the ICU and the hospital. The study offers a straightforward and useful predictor of mortality risk for NCH patients by setting a threshold for PRR.

## 2 Methods

### 2.1 Study population

**Figure 1:**
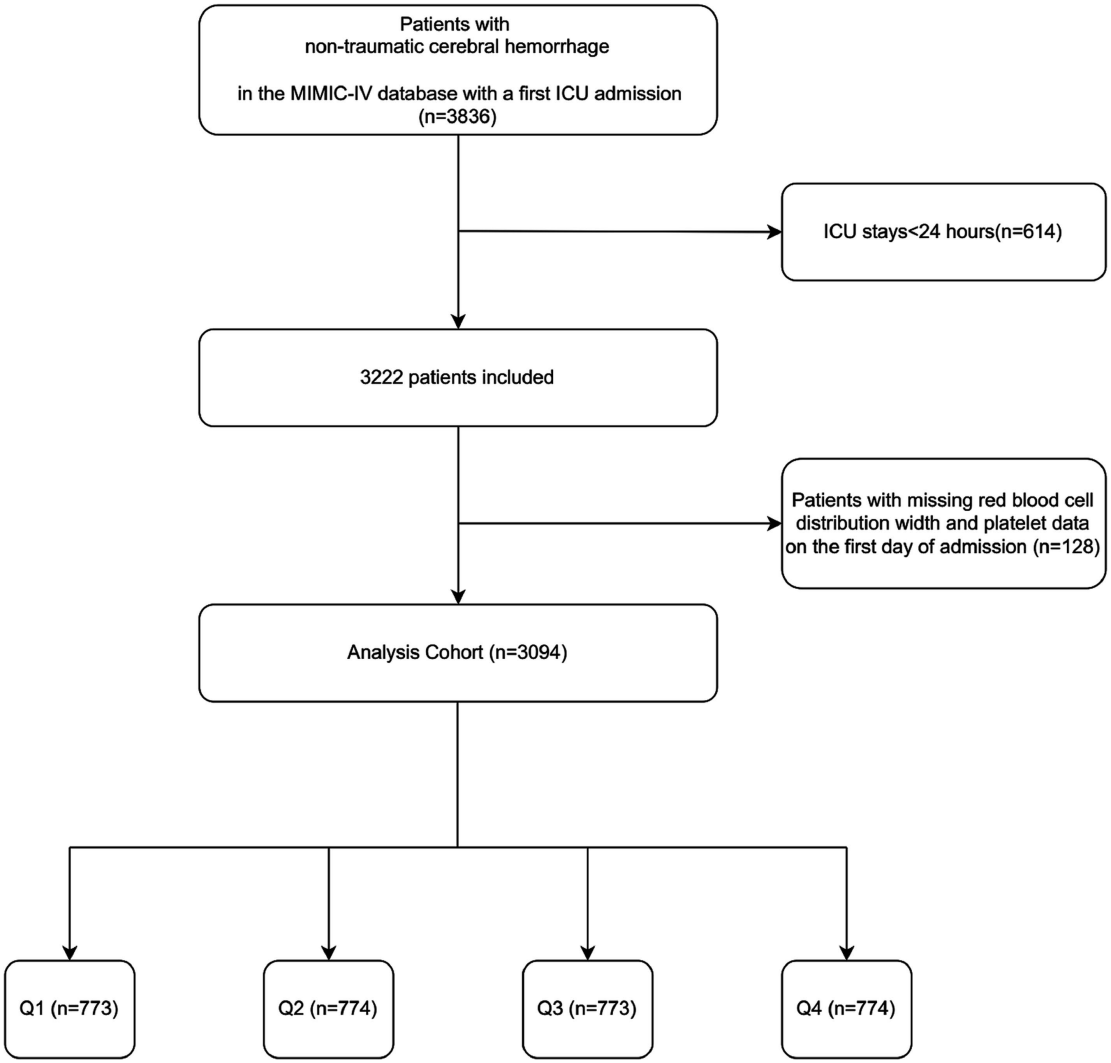
Flowchart of the study cohort.

This retrospective study analyzed health-related data sourced from the MIMIC-IV (version 2.2) database. The MIMIC-IV database, curated and managed by the MIT Computational Physiology Laboratory, is a comprehensive resource that provides extensive clinical data for research purposes. The database contains high-quality medical records of patients admitted to the ICU at Beth Israel Deaconess Medical Center (18). Data extraction was conducted by Changcai Wu, who met the database access requirements (certification number: 41007063). This study extracted 3,836 NCH patients with the first ICU admission. The following patients met the exclusion criteria: (1) those who had been in the ICU for less than 24 h; (2) those who had missing information (platelets and RDW) on the first day of admission. Ultimately, 3,094 patients were enrolled in this study and categorized into four groups according to PRR quartiles (Figure 1). The study was conducted in accordance with the Helsinki Declaration.

### 2.2 Data collection

For this study, data collection was conducted using PostgreSQL and Navicat Premium software. Five groups comprised the variables that were chosen for analysis: (1) demographic factors including age, gender, race, BMI, alcohol abuse, tobacco use, long-term use of antiplatelet agents/anticoagulants; (2) clinical information encompassing systolic blood pressure (SBP), diastolic blood pressure (DBP), respiration rate, temperature, oxygen saturation, hemorrhage site, length of stay in hospital and ICU, ICU and hospital mortality. (3) A number of scoring schemes, including Charlson Comorbidity Index (CCI), Oxford Acute Severity of Illness Score (OASIS), Sequential Organ Failure Score (SOFA), the Glasgow Score Scale (GCS); (4) Comorbidities encompassing congestive heart failure, respiratory failure, diabetes, renal disease, sepsis, malignant cancer, and severe liver disease. (5) Laboratory indicators including hemoglobin, red blood cells (RBC), white blood cells (WBC), platelets, RDW, blood urea nitrogen (BUN), serum creatinine, fasting blood glucose (FBG), serum sodium, serum potassium, international normalized ratio (INR), prothrombin time (PT), partial thromboplastin time (PTT).

The follow-up period began at admission and ended upon the patient’s death or discharge, depending on their outcome. PRR was calculated using platelet count (K/μL) divided by the RDW. Variables with missing values exceeding 20% were not included in this study in order to reduce potential bias. Multiple interpolation was performed for variables with fewer than 20% missing data using the statistical package R 4.2.2 and the Free Statistics software version 1.9.

### 2.3 Clinical outcomes

The primary endpoint of the study was all-cause in-hospital death, while the secondary endpoint was ICU death.

### 2.4 Statistical analysis

Depending on the data distribution, categorical variables were stated as proportions, and continuous variables were provided as mean ± SD or median (interquartile range). The *t*-test or ANOVA was employed for normally distributed continuous variables, whereas the Mann–Whitney *U* test or Kruskal–Wallis test was used for non-normally distributed ones. The incidence of endpoints across various PRR levels was assessed using the log-rank test and Kaplan–Meier survival analysis. Binary logistic regression analysis was employed to evaluate factors associated with all-cause mortality. After accounting for pertinent covariates, hazard ratios (HR) and 95% confidence intervals (CI) for the connection between PRR and outcomes were determined using Cox proportional hazards models. Confounders were selected according to univariate analyses with *p*-values <0.05, as well as clinically and prognostically relevant variables. Three multivariable models were constructed: model 1 (uncorrected), model 2 (adjusted for age, sex, BMI, and race), and model 3 (selected based on various factors). Non-linear associations between baseline PRR and outcomes were assessed using a four-node restricted cubic spline regression model. ROC curve analyses were conducted to determine cut-off values for PRR. P for trends was calculated using interquartile levels. Stratified analyses were carried out to assess the consistent prognostic value of PRR for the outcome. Interaction between PRR and stratification variables was examined using likelihood ratio tests. A *p*-value of less than 0.05 was considered statistically significant. All statistical analyses were performed using R 4.2.2 and Free Statistics software version 1.9.

## 3 Results

In this study, 3,094 critically sick NCH patients were enrolled. The mean age of the patients was 64.9 ± 15.7 years, and 54.0% (1,672) were male. The overall mean PRR was 16.4 ± 6.7. In-hospital mortality was 16.5%, while ICU mortality was 11.8% (Table 1).

**Table 1:** Characteristics and outcomes of participants categorized by PRR*.

Variables	Total (*n* = 3,094)	Q1 (*n* = 773)	Q2 (*n* = 774)	Q3 (*n* = 773)	Q4 (*n* = 774)	*P*-value
Age (years)	64.9 ± 15.7	66.9 ± 15.2	67.3 ± 15.1	64.0 ± 15.8	61.6 ± 16.2	<0.001
Gender: male	1,672 (54.0)	469 (60.7)	455 (58.8)	383 (49.5)	365 (47.2)	<0.001
BMI (kg/m^2^)	27.7 ± 6.5	28.3 ± 6.7	27.8 ± 6.9	27.8 ± 6.3	27.1 ± 6.0	0.092
Race, *n* (%)						0.013
Asian	118 (3.8)	29 (3.8)	37 (4.8)	27 (3.5)	25 (3.2)	
Black	293 (9.5)	96 (12.4)	59 (7.6)	57 (7.4)	81 (10.5)	
White	1923 (62.2)	462 (59.8)	474 (61.2)	495 (64)	492 (63.6)	
Other	760 (24.6)	186 (24.1)	204 (26.4)	194 (25.1)	176 (22.7)	
Site, *n* (%)						<0.001
Cerebellum	60 (1.9)	16 (2.1)	19 (2.5)	13 (1.7)	12 (1.6)	
Cortical	300 (9.7)	77 (10)	79 (10.2)	82 (10.6)	62 (8)	
Intraventricular	112 (3.6)	35 (4.5)	37 (4.8)	18 (2.3)	22 (2.8)	
Subarachnoid	857 (27.7)	163 (21.1)	192 (24.8)	255 (33)	247 (31.9)	
Subdural	426 (13.8)	147 (19)	94 (12.1)	82 (10.6)	103 (13.3)	
Other	1,339 (43.3)	335 (43.3)	353 (45.6)	323 (41.8)	328 (42.4)	
Alcohol abuse	170 (5.5)	57 (7.4)	32 (4.1)	37 (4.8)	44 (5.7)	0.032
Tobacco use	482 (15.6)	132 (17.1)	125 (16.1)	110 (14.2)	115 (14.9)	0.41
SBP (mmHg)	158.2 ± 22.0	155.7 ± 22.2	158.2 ± 22.7	159.7 ± 21.2	159.3 ± 21.9	0.002
DBP (mmHg)	92.1 ± 19.5	91.1 ± 19.5	92.2 ± 19.1	92.1 ± 20.4	93.1 ± 18.9	0.277
Respiratory rate (beats/min)	26.8 ± 6.0	27.7 ± 6.6	26.5 ± 5.4	26.1 ± 5.6	26.8 ± 6.1	<0.001
Temperature (°C)	37.5 ± 0.7	37.5 ± 0.7	37.5 ± 0.7	37.5 ± 0.6	37.5 ± 0.7	0.488
SpO2 (%)	99.6 ± 0.9	99.6 ± 0.9	99.6 ± 0.9	99.6 ± 0.9	99.5 ± 0.9	0.954
Hemoglobin (g/dL)	12.6 ± 2.1	11.7 ± 2.2	12.8 ± 1.8	12.9 ± 1.9	13.0 ± 2.1	<0.001
RBC (m/μL)	3.9 ± 0.7	3.6 ± 0.8	4.0 ± 0.6	4.0 ± 0.7	4.0 ± 0.7	<0.001
WBC (K/μL)	11.8 (8.7, 15.6)	9.4 (7.0, 13.8)	11.2 (8.7, 14.4)	12.3 (9.5, 15.6)	13.7 (10.5, 18.0)	<0.001
Platelets (K/μL)	228.6 ± 93.9	130.7 ± 38.8	195.5 ± 23.7	244.9 ± 27.6	343.3 ± 91.3	<0.001
RDW	14.2 ± 1.8	15.4 ± 2.3	14.0 ± 1.4	13.8 ± 1.3	13.7 ± 1.5	<0.001
BUN (mg/dL)	17.0 (13.0, 24.0)	20.0 (15.0, 30.0)	18.0 (13.0, 23.0)	17.0 (13.0, 22.0)	16.0 (12.0, 22.0)	<0.001
Creatinine (mg/dL)	0.9 (0.8, 1.2)	1.0 (0.8, 1.4)	0.9 (0.8, 1.2)	0.9 (0.7, 1.1)	0.9 (0.7, 1.1)	<0.001
FBG (mg/dL)	143.0 (118.0, 178.0)	138.0 (116.0, 180.0)	141.0 (116.0, 177.0)	144.0 (119.0, 173.0)	148.0 (123.0, 183.8)	0.007
Sodium (mEq/L)	141.1 ± 5.1	141.1 ± 5.5	141.2 ± 5.0	141.4 ± 4.6	140.9 ± 5.2	0.153
Potassium (mEq/L)	4.3 ± 0.8	4.3 ± 0.8	4.3 ± 0.7	4.3 ± 0.8	4.4 ± 0.8	0.008
INR	1.2 (1.1, 1.3)	1.3 (1.1, 1.6)	1.1 (1.1, 1.3)	1.1 (1.1, 1.2)	1.1 (1.1, 1.2)	<0.001
PT (sec)	12.8 (11.8, 14.8)	14.2 (12.5, 17.6)	12.6 (11.7, 14.4)	12.5 (11.6, 13.7)	12.6 (11.7, 13.9)	<0.001
PTT (sec)	29.1 (26.2, 33.6)	31.2 (27.3, 38.2)	28.7 (26.1, 32.8)	28.6 (25.9, 32.0)	28.7 (25.8, 32.4)	<0.001
Congestive heart failure	422 (13.6)	150 (19.4)	115 (14.9)	90 (11.6)	67 (8.7)	<0.001
Respiratory failure	866 (28.0)	289 (37.4)	202 (26.1)	185 (23.9)	190 (24.5)	<0.001
Diabetes	698 (22.6)	198 (25.6)	179 (23.1)	163 (21.1)	158 (20.4)	0.064
Renal disease	381 (12.3)	155 (20.1)	95 (12.3)	65 (8.4)	66 (8.5)	<0.001
Sepsis	1,395 (45.1)	438 (56.7)	321 (41.5)	299 (38.7)	337 (43.5)	<0.001
Malignant cancer	306 (9.9)	129 (16.7)	62 (8)	50 (6.5)	65 (8.4)	<0.001
Severe liver disease	80 (2.6)	72 (9.3)	3 (0.4)	4 (0.5)	1 (0.1)	<0.001
CCI	6.0 (4.0, 8.0)	7.0 (5.0, 9.0)	6.0 (4.0, 7.0)	5.0 (4.0, 7.0)	5.0 (3.0, 7.0)	<0.001
OASIS	32.6 ± 9.0	34.4 ± 9.2	32.4 ± 8.6	31.7 ± 8.8	31.9 ± 9.1	<0.001
SOFA score	3.0 (2.0, 4.0)	3.0 (2.0, 5.0)	2.0 (2.0, 3.0)	2.0 (2.0, 3.0)	2.0 (2.0, 3.0)	<0.001
GCS	11.0 ± 3.9	10.7 ± 4.1	11.2 ± 3.7	11.1 ± 3.7	11.0 ± 3.9	0.058
Long-term use of antiplatelet agents/anticoagulants	849 (27.4)	265 (34.3)	251 (32.4)	181 (23.4)	152 (19.6)	<0.001
Hospital stays (days)	8.8 (4.8, 15.4)	9.5 (4.7, 16.7)	7.9 (4.8, 14.6)	8.7 (4.8, 14.3)	9.0 (5.1, 15.8)	0.027
Hospital mortality	510 (16.5)	187 (24.2)	115 (14.9)	103 (13.3)	105 (13.6)	<0.001
ICU stays (days)	3.8 (2.0, 7.9)	3.5 (1.9, 7.2)	3.6 (2.0, 7.7)	4.0 (2.0, 8.0)	3.8 (2.0, 8.4)	0.164
ICU mortality	366 (11.8)	133 (17.2)	81 (10.5)	72 (9.3)	80 (10.3)	<0.001
PRR	16.4 ± 6.7	8.7 ± 2.7	14.0 ± 1.1	17.8 ± 1.2	25.0 ± 5.6	<0.001

*PRR: Q1(0.71–12.09), Q2(12.10–15.89), Q3(15.90–20.07), Q4(20.08–75.04).

PRR, platelet to red cell distribution width ratio; BMI, body mass index; SBP, systolic blood pressure; DBP, diastolic blood pressure; SpO2, percutaneous oxygen saturation; RBC, red blood cell; WBC, white blood cell; RDW, red cell distribution width; BUN, blood urea nitrogen; FBG, fasting blood glucose; INR, international normalized ratio; PT, prothrombin time; PTT, partial thromboplastin time; CCI, Charlson Comorbidity Index; OASIS, Oxford Acute Severity of Illness Score; SOFA, Sequential organ function score; GCS, Glasgow coma scale; ICU, Intensive Care Unit.

### 3.1 Baseline characteristics

Table 1 displays the baseline characteristics of critically sick patients with NCH, categorized by PRR quartiles. The patients were divided into four groups: Q1 (0.71–12.09), Q2 (12.10–15.89), Q3 (15.90–20.07), and Q4 (20.08–75.04). The mean PRR values for each quartile were 8.7 ± 2.7, 14.0 ± 1.1, 17.8 ± 1.2, and 25.0 ± 5.6, respectively. Hemoglobin, WBC, platelets, FBG, and potassium levels were generally greater in patients in the highest PRR quartile than those in the lower quartiles. Additionally, younger age, a greater percentage of female patients, lower BMI, RDW, and BUN levels, along with a decreased incidence of severe liver disease, congestive heart failure, and long-term antiplatelet/anticoagulant use were all linked to higher PRR values.

Table 2 lists the initial features of survivors and non-survivors during hospitalization. Congestive heart failure, respiratory failure, renal disease, sepsis, and severe liver disease were more common in the non-survivor group of patients, who were also older and had higher SBP, respiratory rates, and temperatures. Additionally, non-survivors had elevated levels of WBC, RDW, BUN, creatinine, FBG, sodium, potassium, INR, PT, CCI, OASIS, and SOFA scores. Notably, the non-survivor group had substantially lower PRR levels than the survivor group (14.1 vs. 16.1, *p* < 0.001).

**Table 2:** Baseline characteristics of the survivors and non-survivors groups.

Variables	Total (*n* = 3,094)	Survivor (*n* = 2,584)	Non-survivor (*n* = 510)	*P*-value
Age (years)	64.9 ± 15.7	64.2 ± 15.8	68.8 ± 15.0	<0.001
Gender: male	1,672 (54.0)	1,409 (54.5)	263 (51.6)	0.22
BMI (kg/m^2^)	27.7 ± 6.5	27.8 ± 6.3	27.6 ± 7.1	0.626
Race, *n* (%)				<0.001
Asian	118 (3.8)	97 (3.8)	21 (4.1)	
Black	293 (9.5)	253 (9.8)	40 (7.8)	
White	1923 (62.2)	1,675 (64.8)	248 (48.6)	
Other	760 (24.6)	559 (21.6)	201 (39.4)	
Site, *n* (%)				0.005
Cerebellum	60 (1.9)	51 (2)	9 (1.8)	
Cortical	300 (9.7)	261 (10.1)	39 (7.6)	
Intraventricular	112 (3.6)	88 (3.4)	24 (4.7)	
Subarachnoid	857 (27.7)	704 (27.2)	153 (30)	
Subdural	426 (13.8)	379 (14.7)	47 (9.2)	
Other	1,339 (43.3)	1,101 (42.6)	238 (46.7)	
Alcohol abuse	170 (5.5)	139 (5.4)	31 (6.1)	0.527
Tobacco use	482 (15.6)	416 (16.1)	66 (12.9)	0.072
SBP (mmHg)	158.2 ± 22.0	157.5 ± 22.0	162.0 ± 22.1	<0.001
DBP (mmHg)	92.1 ± 19.5	92.4 ± 19.5	90.8 ± 19.2	0.107
Respiratory rate (beats/min)	26.8 ± 6.0	26.6 ± 5.9	27.8 ± 6.2	<0.001
Temperature (°C)	37.5 ± 0.7	37.5 ± 0.6	37.8 ± 0.9	<0.001
SpO2 (%)	99.6 ± 0.9	99.5 ± 0.9	99.7 ± 0.9	<0.001
Hemoglobin (g/dL)	12.6 ± 2.1	12.6 ± 2.0	12.5 ± 2.2	0.228
RBC (m/μL)	3.9 ± 0.7	3.9 ± 0.7	3.9 ± 0.8	0.019
WBC (K/μL)	11.8 (8.7, 15.6)	11.4 (8.6, 15.1)	13.9 (10.3, 17.7)	<0.001
Platelets (K/μL)	217.5 (170.0, 273.0)	220.0 (175.0, 274.0)	202.5 (148.0, 265.0)	<0.001
RDW	14.2 ± 1.8	14.1 ± 1.7	14.7 ± 2.0	<0.001
BUN (mg/dL)	17.0 (13.0, 24.0)	17.0 (13.0, 23.0)	21.0 (16.0, 31.0)	<0.001
Creatinine (mg/dL)	0.9 (0.8, 1.2)	0.9 (0.7, 1.2)	1.0 (0.8, 1.5)	<0.001
FBG (mg/dL)	143.0 (118.0, 178.0)	138.0 (116.0, 170.0)	170.5 (137.0, 223.8)	<0.001
Sodium (mEq/L)	141.1 ± 5.1	140.6 ± 4.3	143.5 ± 7.4	<0.001
Potassium (mEq/L)	4.3 ± 0.8	4.3 ± 0.7	4.5 ± 0.9	<0.001
INR	1.2 (1.1, 1.3)	1.1 (1.1, 1.3)	1.2 (1.1, 1.4)	<0.001
PT (sec)	12.8 (11.8, 14.8)	12.7 (11.7, 14.5)	13.5 (12.3, 15.8)	<0.001
PTT (sec)	29.1 (26.2, 33.6)	29.0 (26.2, 33.4)	29.8 (26.2, 35.0)	0.052
Congestive heart failure	422 (13.6)	328 (12.7)	94 (18.4)	<0.001
Respiratory failure	866 (28.0)	615 (23.8)	251 (49.2)	<0.001
Diabetes	698 (22.6)	569 (22)	129 (25.3)	0.106
Renal disease	381 (12.3)	296 (11.5)	85 (16.7)	0.001
Sepsis	1,395 (45.1)	1,092 (42.3)	303 (59.4)	<0.001
Malignant cancer	306 (9.9)	259 (10)	47 (9.2)	0.577
Severe liver disease	80 (2.6)	53 (2.1)	27 (5.3)	<0.001
CCI	6.0 (4.0, 8.0)	5.0 (4.0, 7.0)	7.0 (5.0, 8.0)	<0.001
OASIS	32.6 ± 9.0	31.1 ± 8.4	40.3 ± 8.1	<0.001
SOFA score	3.0 (2.0, 4.0)	2.0 (2.0, 4.0)	3.0 (2.0, 4.0)	<0.001
GCS	13.0 (8.0, 14.0)	13.0 (9.0, 14.0)	7.0 (3.0, 14.0)	<0.001
Long-term use of antiplatelet agents/anticoagulants	849 (27.4)	754 (29.2)	95 (18.6)	<0.001
PRR	15.9 (12.1, 20.1)	16.1 (12.5, 20.2)	14.1 (10.1, 18.6)	<0.001

PRR, platelet to red cell distribution width ratio; BMI, body mass index; SBP, systolic blood pressure; DBP, diastolic blood pressure; SpO2, percutaneous oxygen saturation; RBC, red blood cell; WBC, white blood cell; RDW, red cell distribution width; BUN, blood urea nitrogen; FBG, fasting blood glucose; INR, international normalized ratio; PT, prothrombin Time; PTT, partial thromboplastin time; CCI, Charlson Comorbidity Index; OASIS, Oxford Acute Severity of Illness Score; SOFA, sequential organ function score; GCS, Glasgow coma scale.

### 3.2 Outcomes

In critically sick patients with NCH, binary logistic regression was used to examine the risk of all-cause death. Additionally, prognostic indicators based on clinician recommendations and clinical experience were analyzed as independent variables, and the results are shown in Supplementary Table S1. The results revealed that age, race, site, SBP, respiratory rate, temperature, SpO2, RBC, WBC, BUN, creatinine, FBG, sodium, potassium, INR, PT, PTT, congestive heart failure, respiratory failure, renal disease, sepsis, severe liver disease, CCI, OASIS, sofa score, GCS, long-term use of antiplatelet agents/anticoagulants, and PRR were significant influencing factors.

**Figure 2:**
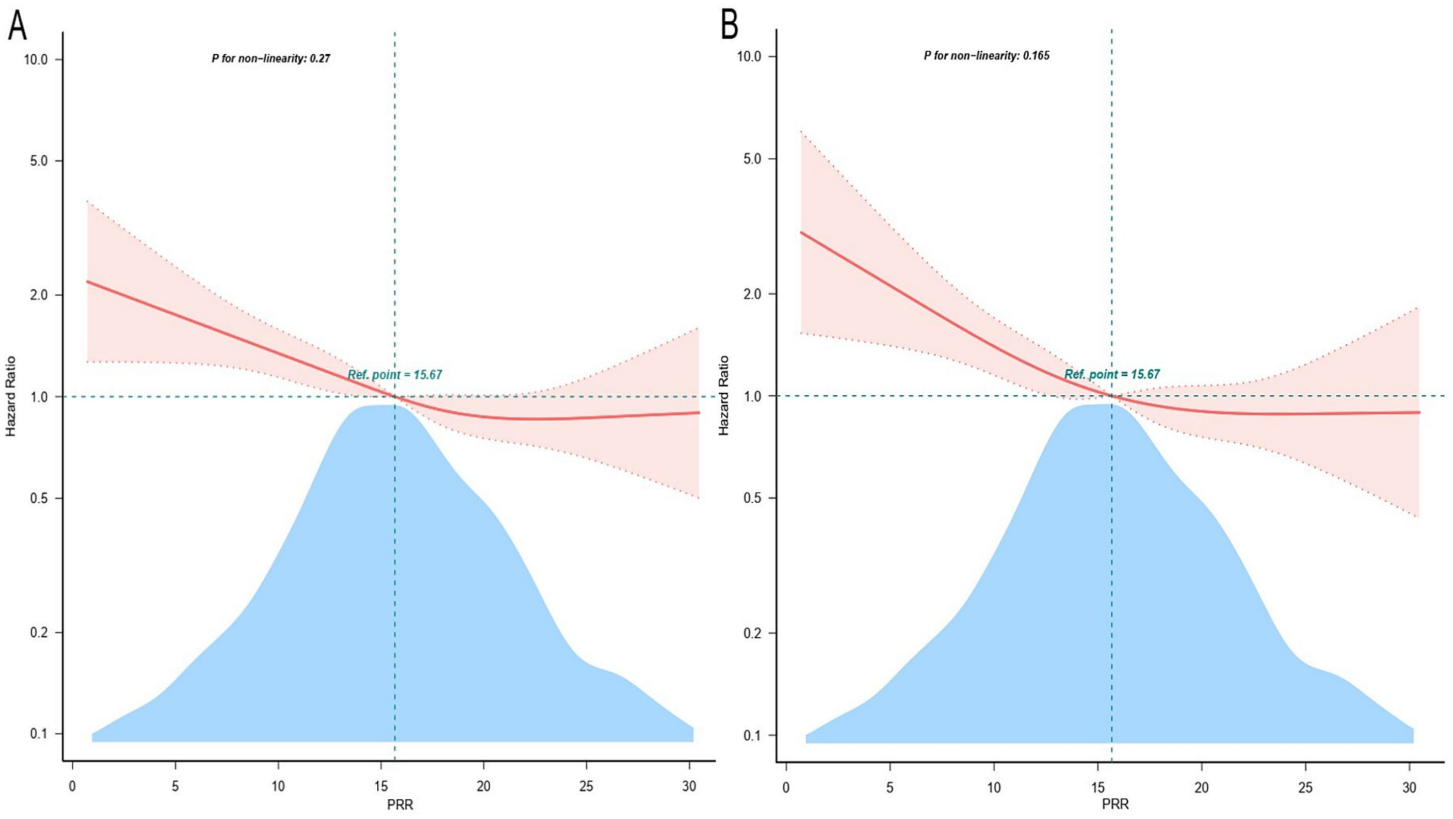
Restricted cubic spline curve for the PRR hazard ratio. Adjustment for age, gender, BMI, race, site, SBP, respiratory rate, temperature, SpO2, RBC, WBC, BUN, creatinine, FBG, sodium, potassium, INR, PT, PTT, congestive heart failure, respiratory failure, renal disease, sepsis, severe liver disease, CCI, OASIS, sofa score, GCS, long-term use of antiplatelet agents/anticoagulants. The red solid line and the light red shadow represent the estimated values and their corresponding 95% confidence intervals, respectively. **(A)** Restricted cubic spline for hospital mortality. **(B)** Restricted cubic spline for ICU mortality. HR, hazard ratio; CI, confidence interval; ICU, intensive care unit; PRR, platelet-to-red cell distribution width ratio.

Furthermore, to explore the connection between PRR and hospital death, a Cox proportional hazards analysis was conducted. When PRR was viewed as a continuous variable, the results showed that low PRR was a substantially risk indicator in all three models: the uncorrected model (HR, 0.97; 95% CI, 0.96–0.99; *p* < 0.001), the partially corrected model (HR, 0.97; 95% CI, 0.96–0.99; *p* < 0.001), and the fully corrected model (HR, 0.98; 95% CI, 0.96–0.99; *p* = 0.006). Our findings indicated that for every 1-unit increase in PRR, the risk of hospital death reduces by 2%. When PRR was regarded a categorical variable, the risk of in-hospital death was notably lower in patients in the higher PRR quartiles across three established Cox proportional risk models: uncorrected model (HR, 0.60; 95% CI, 0.47–0.76; *p* < 0.001), partially corrected model (HR, 0.67; 95% CI, 0.52–0.85; *p* = 0.001), and fully corrected models (HR, 0.67; 95% CI, 0.51–0.88; *p* = 0.004), compared with subjects in the lowest quartile (Table 3). Considering comparable risks of in-hospital death, Q2, Q3, and Q4 were combined into a single group (Q2-4, PRR >12.09), showing significantly lower in-hospital mortality compare to Q1 (Supplementary Table S2). Table 3 and Supplementary Table S2 displays comparable findings from multivariate Cox proportional risk models for PRR and ICU mortality. Additionally, the probability of hospital and ICU mortality decreased linearly with increasing PRR when restricted cubic spline regression models were used (non-linear *p* = 0.270, non-linear *p* = 0.165) (Figure 2).

**Table 3:** Cox proportional hazard ratios (HR) for all-cause mortality.

Categories	Model 1			Model 2			Model 3	
	HR (95% CI)	*P*-value		HR (95% CI)	*P*-value		HR (95% CI)	*P*-value
Hospital mortality								
Continuous variable per unit	0.97 (0.96–0.99)	<0.001		0.97 (0.96–0.99)	<0.001		0.98 (0.96–0.99)	0.006
Quartile*								
Q1 (*n* = 773)	Ref			Ref			Ref	
Q2 (*n* = 774)	0.69 (0.55–0.87)	0.002		0.65 (0.51–0.82)	<0.001		0.73 (0.57–0.94)	0.013
Q3 (*n* = 773)	0.62 (0.49–0.79)	<0.001		0.61 (0.47–0.77)	<0.001		0.63 (0.48–0.82)	0.001
Q4 (*n* = 774)	0.60 (0.47–0.76)	<0.001		0.67 (0.52–0.85)	0.001		0.67 (0.51–0.88)	0.004
*P* for trend		<0.001			<0.001			0.002
ICU mortality								
Continuous variable per unit	0.96 (0.95–0.98)	<0.001		0.97 (0.95–0.99)	0.001		0.98 (0.96–0.99)	0.027
Quartile*								
Q1 (*n* = 773)	Ref			Ref			Ref	
Q2 (*n* = 774)	0.59 (0.44–0.77)	<0.001		0.57 (0.43–0.76)	<0.001		0.67 (0.50–0.90)	0.008
Q3 (*n* = 773)	0.50 (0.38–0.67)	<0.001		0.51 (0.38–0.68)	<0.001		0.58 (0.42–0.79)	0.001
Q4 (*n* = 774)	0.55 (0.42–0.73)	<0.001		0.65 (0.49–0.86)	0.003		0.65 (0.47–0.89)	0.008
*P* for trend		<0.001			<0.001			0.004

*PRR: Q1(0.71–12.09), Q2(12.10–15.89), Q3(15.90–20.07), Q4(20.08–75.04).

Model 1: unadjusted.

Model 2: adjusted for age, gender, BMI, race.

Model 3: adjusted for age, gender, BMI, race, site, SBP, respiratory rate, temperature, SpO2, RBC, WBC, BUN, creatinine, FBG, sodium, potassium, INR, PT, PTT, congestive heart failure, respiratory failure, renal disease, sepsis, severe liver disease, CCI, OASIS, sofa score, GCS, long-term use of antiplatelet agents/anticoagulants.

**Figure 3:**
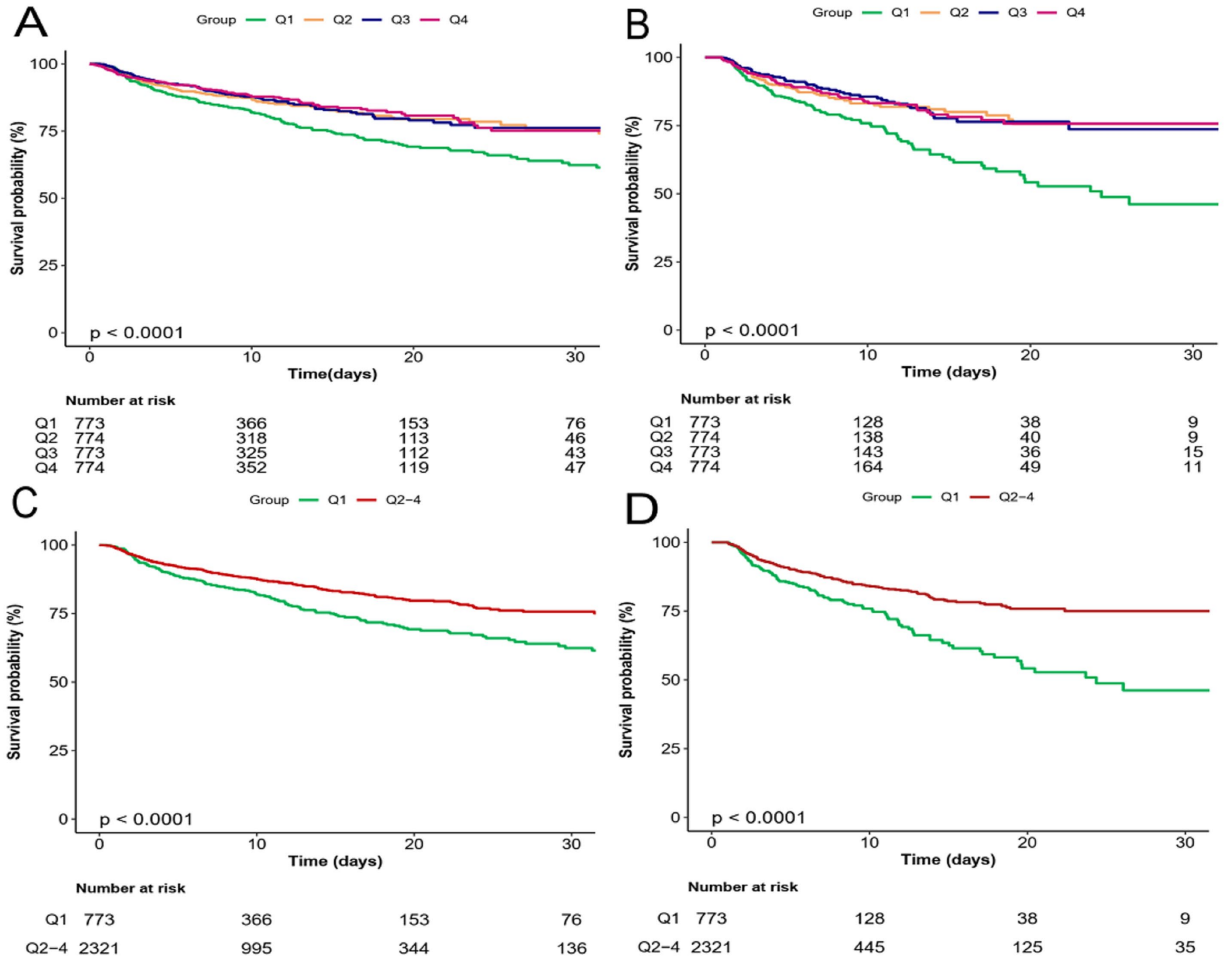
Kaplan–Meier survival analysis curves for all-cause mortality. Kaplan–Meier curves showing cumulative probability of all-cause mortality according to groups at 30 days. **(A,C)** Kaplan–Meier survival analysis curves for hospital mortality. **(B,D)** Kaplan–Meier survival analysis curves for ICU mortality.

To analyze the incidence of outcomes across PRR quartiles, the study employed Kaplan–Meier survival curves, as demonstrated in Figure 3. Individuals in the lower PRR quartile showed an increased probability of death in both the ICU and hospital. ROC analysis was used to assess the clinical efficacy of PRR; however, the AUC for PRR was not at desirable levels (AUC for in-hospital death: 0.585 [0.556, 0.613]; AUC for ICU death: 0.570 [0.537, 0.603]) (Supplemental Figure S1). The PRR cut-off value for predicting both in-hospital and ICU death was determined to be 11.16.

### 3.3 Subgroup analysis

**Figure 4:**
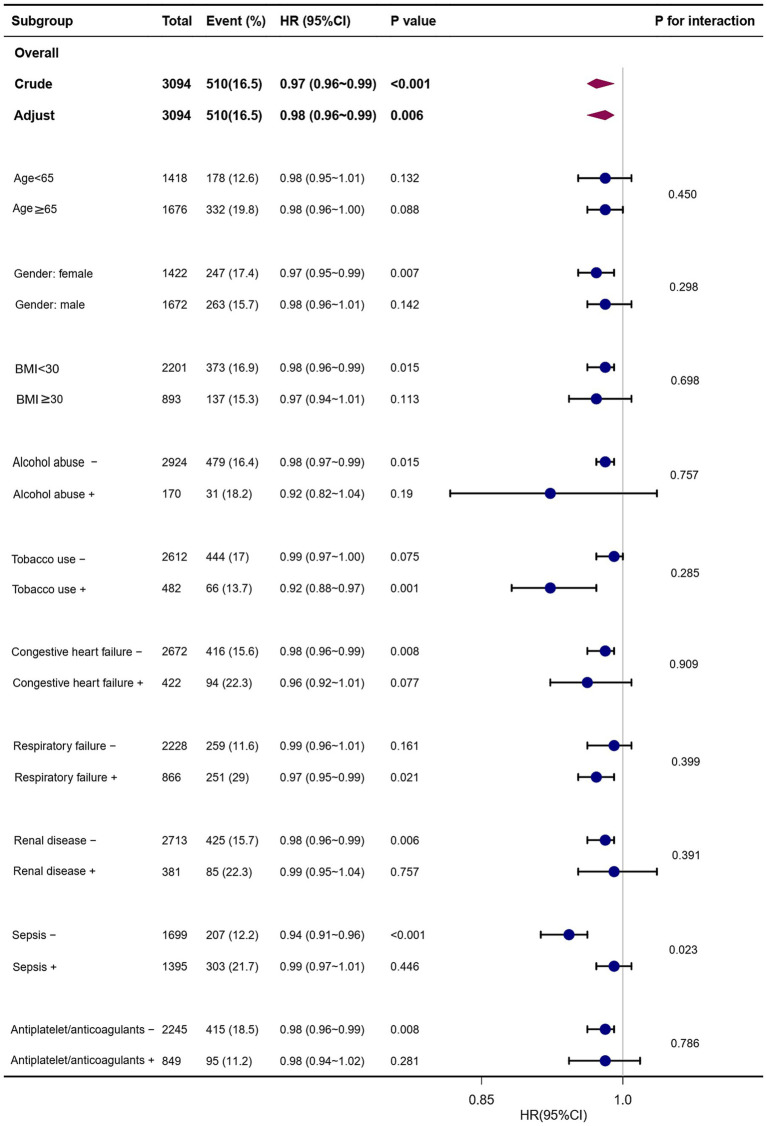
Forest plots of hazard ratios for the hospital mortality in different subgroups. HR, hazard ratio; CI, confidence interval; BMI, body mass index.

Several patient subgroups were examined to determine the risk stratification utility of PRR for outcomes, including age, gender, BMI, alcohol abuse, tobacco use, congestive heart failure, respiratory failure, renal disease, sepsis, and long-term use of antiplatelet/anticoagulants. Among the NCH patient subgroups, the PRR was substantially associated with a lower risk of hospital mortality: females (HR, 0.97; 95% CI, 0.95–0.99), those with BMI <30 kg/m^2^ (HR, 0.98; 95% CI, 0.96–0.99), those who do not abuse alcohol (HR, 0.98; 95% CI, 0.97–0.99), tobacco users (HR, 0.92; 95% CI, 0.88–0.97), those without congestive heart failure (HR, 0.98; 95% CI, 0.96–0.99), those with respiratory failure (HR, 0.97; 95% CI, 0.95–0.99), individuals without renal disease (HR, 0.98; 95% CI, 0.96–0.99), those without sepsis (HR, 0.94; 95% CI, 0.91–0.96), and patients not receiving long-term antiplatelet/anticoagulant therapy (HR, 0.98; 95% CI, 0.96–0.99) (Figure 4).

**Figure 5:**
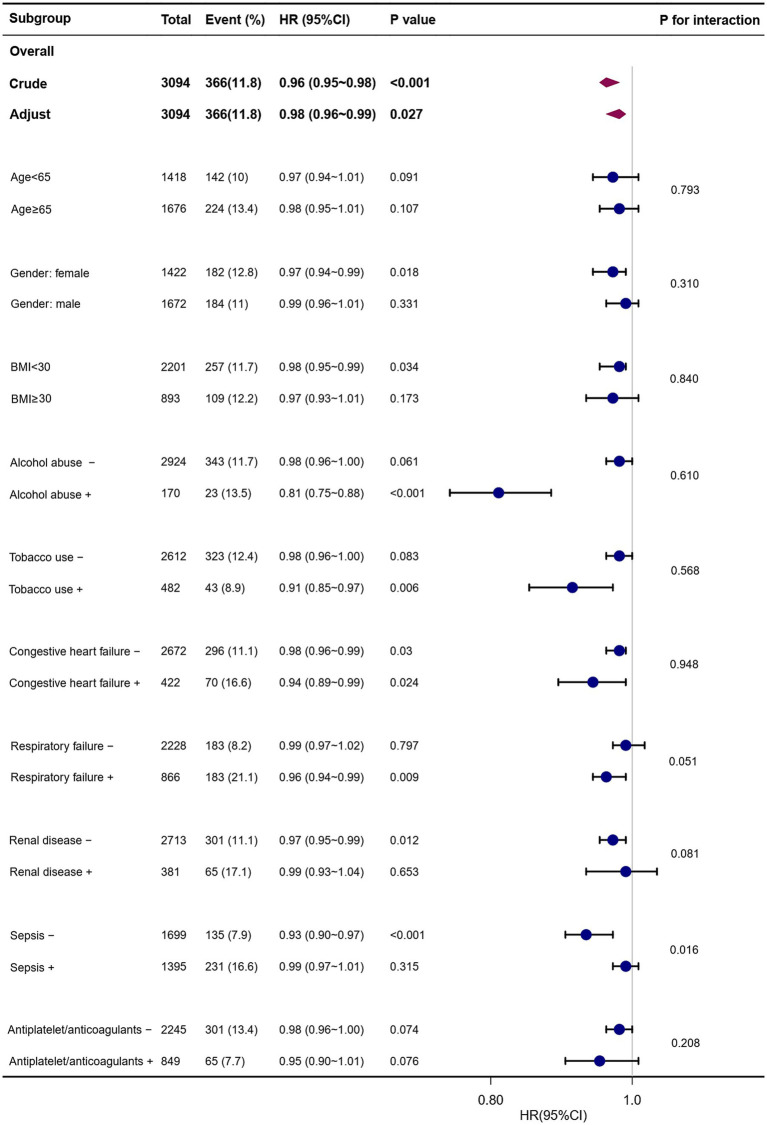
Forest plots of hazard ratios for the ICU mortality in different subgroups. HR, hazard ratio; CI, confidence interval; BMI, body mass index.

Similarly, PRR showed a significant correlation with a lower risk of ICU mortality in the following subgroups: those without congestive heart failure (HR, 0.98; 95% CI, 0.96–0.99), those with congestive heart failure (HR, 0.94; 95% CI, 0.89–0.99), females (HR, 0.97; 95% CI, 0.94–0.99), those with BMI <30 kg/m^2^ (HR, 0.98; 95% CI, 0.95–0.99), those abusing alcohol (HR, 0.81; 95% CI, 0.75–0.88), those who use tobacco (HR, 0.91; 95% CI, 0.85–0.97), those with respiratory failure (HR, 0.96; 95% CI, 0.94–0.99), those without renal disease (HR, 0.97; 95% CI, 0.95–0.99), and those without sepsis (HR, 0.93; 95% CI, 0.90–0.97) (Figure 5).

Interestingly, the prognostic significance of PRR seems to be more prominent in patients without sepsis [(HR, 0.94; 95% CI, 0.91–0.96) vs. with sepsis (HR, 0.99; 95% CI, 0.97–1.01); *P* for interaction = 0.023] (Figure 4).

## 4 Discussion

In this study, we investigated the association between PRR and clinical outcomes in a US cohort of critically ill NCH patients. Our findings suggest that in these patients, a greater PRR was associated with a lower probability of all-cause mortality in the hospital and ICU. When confounding risk factors were taken into account, the connection between PRR and all-cause ICU and hospital mortality remained robust. Hence, PRR may be useful to physicians in making decisions and could independently serve as a risk factor for critically ill patients with NCH.

Lower PRR levels in non-survivors, compared to those who survived, typically indicate lower platelet counts and higher RDW. A previous study suggested that RDW could serve as a reliable and independent indicator of 30-day mortality in NCH patients (19). However, the mechanism underlying the correlation between increased RDW and adverse outcomes is not fully understood. Several plausible explanations can be considered. First, elevated RDW may signify the presence of systemic inflammation, as evidenced by its correlation with common inflammatory markers like C-reactive protein and erythrocyte sedimentation rate (20). Inflammatory cytokines, such as TNF-*α*, IL-1β, and IL-6, inhibit erythropoietin production, leading to immature reticulocytes being released into the peripheral circulation (21–23). This, in turn, contributes to an elevated RDW. Second, oxidative stress can also hinder erythropoiesis and impair the deformability of erythrocyte membranes, resulting in a shortened lifespan of erythrocytes and an increased RDW (24). In patients with NCH, oxidative stress has been linked to neuronal dysfunction and cell death (25–27). Elevated RDW may, therefore, reflect heightened oxidative stress levels, contributing to poor outcomes in NCH patients. Finally, the sympatho-adrenal and renin-angiotensin systems may stimulate erythropoiesis by upregulating erythropoietin expression (28, 29). Patients with NCH often experience harmful catecholamine surges that adversely affect multiorgan function (30, 31). We hypothesize that high RDW is related with excessive catecholamine and angiotensin II-mediated dysfunction of peripheral organs, leading to increased mortality rates. Platelets play a crucial role in coordinating systemic inflammation and immune responses. P-selectin expression on platelets and subsequent formation of platelet-leukocyte aggregates enhance proinflammatory functions of leukocytes (32). A retrospective observational study, based on a large database, demonstrated that thrombocytopenia and the course of plateletopathies significantly impact in-hospital mortality in patients in neurological ICU (33). Moreover, mounting evidence suggested that platelet dysfunction was linked to mortality in patients with hemorrhagic stroke (34, 35). Cerebral hemorrhage can induce significant inflammatory and oxidative stress, contributing to secondary brain injury and poor prognosis (36). Elevated RDW is typically linked to these conditions, and platelets are key to coagulation and immune response (9). A single index can be influenced by various factors. As a composite measure, the platelet to RDW ratio (PRR) integrates data from both, acting as a potential inflammation marker (14). It reflects the body’s inflammatory load and metabolic state, serving as a predictor of disease severity and helping identify high-risk patients.

The PRR has been considered an effective prognostic factor for various diseases like hepatic fibrosis (37), sepsis (38, 39), myocardial infarction (40), and acute pancreatitis (41). Wu et al. (13) demonstrated that in critically sick patients with acute kidney injury, a reduced PRR was substantially linked to a higher risk of in-hospital mortality. Furthermore, PRR was more effective as an indicator than platelets and RDW (13). By integrating the prognostic benefits of platelets and RDW, PRR served as a novel indicator of the severity of inflammation (14). Similarly, Ge et al. (15) found that the change of PRR level after acute traumatic brain injury was related to the development of inflammatory (15). PRR was straightforward to evaluate, positioning it as a potentially significant predictive marker (42). Research by Xi and Bai (43) demonstrated that PRR provides superior diagnostic value for hemophagocytic lymphohistiocytosis compared to RDW. Additionally, previous research has shown that lower PRR was linked to a high risk of 30-day [HR, 1.45 (95% CI 1.10–1.92), *p* = 0.009] and 1-year mortality [HR, 1.54 (95% CI 1.23–1.93), *p* < 0.001] in acute ischemic stroke (44). Currently, there is limited research on the connection between PRR and clinical outcomes in severely ill NCH patients. Lehmann et al. (17) reported that patients with high PRR had significantly lower 90-day mortality following bleeding events compared to those with low PRR (27% vs. 57%; *p* = 0.003, OR: 3.6, 95%CI: 1.6–8.3). However, this study had a sample size of only 102 patients and primarily focused on individuals with deep-seated intracerebral hemorrhage. In our extensive cohort, lower PRR was found to be a significant independent risk factor for increased mortality among critically ill patients with NCH in the ICU. Rigid statistical techniques were employed to validate the relationship between PRR and ICU and hospital mortality in NCH patients. Clinically, combining PRR with parameters such as blood pressure, oxygen saturation, and imaging results can enhance prognostic accuracy through a comprehensive risk model. In critically ill patients, dynamic PRR monitoring allows timely evaluation of disease progression and treatment efficacy, enabling prompt interventions. For patients with NCH, PRR assists in early risk stratification, identifying high-risk individuals and guiding targeted surveillance and treatment. Changes in PRR can inform adjustments in treatment regimens, such as platelet transfusions or anti-inflammatory therapies, allowing personalized interventions to improve outcomes. Additionally, as RDW and platelet counts are derived from routine, rapid, and cost-effective complete blood count tests, PRR can be easily and continuously monitored in clinical practice.

This study also examined risk stratification among different categories in further detail. Reduced PRR was strongly linked to an increase risk of ICU mortality in individuals with and without congestive heart failure, according to our subgroup analysis. However, in patients with congestive heart failure, renal disease, or sepsis, we did not observe any correlation between PRR and in-hospital all-cause death. Reverse causality may be to blame for this phenomenon: despite a higher risk of all-cause mortality, patients with these comorbidities had a better prognosis because they were more likely to have received the optimal therapy or developed healthier lifestyle choices. Moreover, in this study, patients without sepsis had a higher predictive value of PRR (HR, 0.94; 95% CI, 0.91–0.96) than patients with sepsis (HR, 0.99; 95% CI, 0.97–1.01; *P* for interaction = 0.023), indicating that sepsis treatment may have an impact on PRR’s ability to predict all-cause mortality. It’s possible that this discrepancy results from the increased likelihood of anti-inflammatory medication being given to sepsis patients. Previous studies have shown that inflammatory mechanisms contribute to the progression of NCH-induced secondary brain injury, and inhibiting inflammatory pathways can mitigate secondary brain injury caused by cerebral hemorrhage (45, 46).

The study had several limitations. Firstly, its retrospective approach prevented it from conclusively establishing causation. The clinical results could have been impacted by residual confounding factors even though we employed subgroup analysis and multivariate adjustment. Additionally, this database does not contain information on possible confounders including etiology of the hemorrhage, hemorrhage volumes, neurologic complications, treatment protocols, and nutritional deficiencies. Furthermore, dynamic changes during the hospital and ICU stays were not monitored; only the baseline PRR was examined. Therefore, the predictive significance of PRR alterations should be assessed in future studies. Finally, detailed information on factors like disease severity, functional status at discharge, and social support was unavailable, which could impact mortality. Subsequent studies should consider these factors to strengthen the validation of the association between PRR and mortality.

## 5 Conclusion

In summary, our study extended the applicability of PRR to non-traumatic cerebral hemorrhage patients in critical condition. It was discovered that PRR might be a useful metric for risk categorization of these patients’ ICU and in-hospital mortality. Clinical practice decision-making and disease management may benefit from PRR monitoring. However, further research is required to investigate whether improving control over PRR will ultimately lead to improved clinical prognosis.

## Data Availability

Publicly available datasets were analyzed in this study. This data can be found: MIMIC-VI (https://physionet.org).
