# Supplementary material for: Association between platelet-to-red cell distribution width ratio and all-cause mortality in critically ill patients with non-traumatic cerebral hemorrhage: a retrospective cohort study
Source: Front Neurol. 2024 Nov 28;15:1456884. doi: 10.3389/fneur.2024.1456884 (PMC11634754; doi:10.3389/fneur.2024.1456884)
Supplement: Supplementary file 1 [file Table_1.docx]

**Table S1** Binary logistic regression analysis of the factors influencing all-cause death of the study population

| **Variables** | **OR_95CI** | **P_value** |
| --- | --- | --- |
| Age (years) | 1.02 (1.01~1.03) | <0.001 |
| Gender: male VS. female | 0.89 (0.73~1.07) | 0.221 |
| BMI (kg/m^2^) | 0.99 (0.97~1) | 0.075 |
| Race |  |  |
| Asian | 0.6 (0.37~0.99) | 0.046 |
| Black | 0.44 (0.3~0.64) | <0.001 |
| White | 0.41 (0.33~0.51) | <0.001 |
| Other | Ref |  |
| Site |  |  |
| Cerebellum | 0.82 (0.4~1.68) | 0.582 |
| Cortical | 0.69 (0.48~1) | 0.047 |
| Intraventricular | 1.26 (0.79~2.02) | 0.335 |
| Subarachnoid | 1.01 (0.8~1.26) | 0.963 |
| Subdural | 0.57 (0.41~0.8) | 0.001 |
| Other | Ref |  |
| Alcohol abuse + | 1.14 (0.76~1.7) | 0.527 |
| Tobacco use + | 0.77 (0.59~1.02) | 0.073 |
| SBP (mmHg) | 1.01 (1~1.01) | <0.001 |
| DBP (mmHg) | 1 (0.99~1) | 0.1 |
| Respiratory rate (beats/min) | 1.03 (1.02~1.05) | <0.001 |
| Temperature (°C) | 2.03 (1.78~2.31) | <0.001 |
| SpO2 (%) | 1.45 (1.26~1.68) | <0.001 |
| Hemoglobin (g/dL) | 0.97 (0.93~1.02) | 0.228 |
| RBC (m/uL) | 0.86 (0.75~0.97) | 0.017 |
| WBC (K/uL) | 1.01 (1~1.02) | 0.002 |
| BUN (mg/dL) | 1.02 (1.02~1.03) | <0.001 |
| Creatinine (mg/dL) | 1.15 (1.08~1.22) | <0.001 |
| FBG (mg/dL) | 1.01 (1~1.01) | <0.001 |
| Sodium (mEq/L) | 1.11 (1.09~1.13) | <0.001 |
| Potassium (mEq/L) | 1.34 (1.2~1.49) | <0.001 |
| INR | 1.12 (1.02~1.22) | 0.012 |
| PT (sec) | 1.01 (1~1.02) | 0.004 |
| PTT (sec) | 1.01 (1~1.01) | 0.005 |
| Congestive heart failure + | 1.55 (1.21~2) | 0.001 |
| Respiratory failure + | 3.1 (2.55~3.77) | <0.001 |
| Diabetes + | 1.2 (0.96~1.49) | 0.106 |
| Renal disease + | 1.55 (1.19~2.01) | 0.001 |
| Sepsis + | 2 (1.65~2.43) | <0.001 |
| Malignant cancer + | 0.91 (0.66~1.26) | 0.577 |
| Severe liver disease + | 2.67 (1.66~4.29) | <0.001 |
| CCI | 1.13 (1.1~1.17) | <0.001 |
| OASIS | 1.13 (1.12~1.15) | <0.001 |
| SOFA score | 1.2 (1.14~1.26) | <0.001 |
| GCS | 0.8 (0.79~0.82) | <0.001 |
| Long-term use of antiplatelet agents/anticoagulants | 0.56 (0.44~0.71) | <0.001 |
| PRR | 0.96 (0.94~0.97) | <0.001 |

Abbreviation: PRR, Platelet to red cell distribution width ratio; BMI, body mass index; SBP, systolic blood pressure; DBP, diastolic blood pressure; SpO2, percutaneous oxygen saturation; RBC, red blood cell; WBC, white blood cell; BUN, blood urea nitrogen; FBG, fasting blood glucose; INR, international normalized ratio; PT, prothrombin Time; PTT, partial thromboplastin time; CCI, Charlson Comorbidity Index; OASIS, Oxford Acute Severity of Illness Score; SOFA, Sequential organ function score; GCS, Glasgow coma scale
