# Supplementary material for: Association between platelet-to-red cell distribution width ratio and all-cause mortality in critically ill patients with non-traumatic cerebral hemorrhage: a retrospective cohort study
Source: Front Neurol. 2024 Nov 28;15:1456884. doi: 10.3389/fneur.2024.1456884 (PMC11634754; doi:10.3389/fneur.2024.1456884)
Supplement: Supplementary file 2 [file Table_2.docx]

**Table S2.** Cox proportional hazard ratios (HR) for all-cause mortality

| **Categories** | **Model 1** | |  | **Model 2** | |  | **Model 3** | |
| --- | --- | --- | --- | --- | --- | --- | --- | --- |
|  | **HR (95% CI)** | **P-value** |  | **HR (95% CI)** | **P-value** |  | **HR (95% CI)** | **P-value** |
| **Hospital mortality** |  |  |  |  |  |  |  |  |
| **Group** |  |  |  |  |  |  |  |  |
| Q1 (PRR <12.09) | Ref |  |  | Ref |  |  | Ref |  |
| Q2-4(PRR >12.09) | 0.64 (0.53-0.76) | <0.001 |  | 0.64 (0.53-0.77) | <0.001 |  | 0.68 (0.55-0.84) | <0.001 |
| **ICU mortality** |  |  |  |  |  |  |  |  |
| **Group** |  |  |  |  |  |  |  |  |
| Q1 (PRR <12.09) | Ref |  |  | Ref |  |  | Ref |  |
| Q2-4(PRR >12.09) | 0.55 (0.44-0.68) | <0.001 |  | 0.57 (0.46-0.71) | <0.001 |  | 0.63 (0.49-0.81) | <0.001 |

Model 1: unadjusted

Model 2: adjusted for age, gender, BMI, race

Model 3: adjusted for age, gender, BMI, race, site, SBP, respiratory rate, temperature, SpO2, RBC, WBC, BUN, creatinine, FBG, sodium, potassium, INR, PT, PTT, congestive heart failure, respiratory failure, renal disease, sepsis, severe liver disease, CCI, OASIS, sofa score, GCS, long-term use of antiplatelet/anticoagulants
