# Supplementary material for: Association between platelet-to-red cell distribution width ratio and all-cause mortality in critically ill patients with non-traumatic cerebral hemorrhage: a retrospective cohort study
Source: Front Neurol. 2024 Nov 28;15:1456884. doi: 10.3389/fneur.2024.1456884 (PMC11634754; doi:10.3389/fneur.2024.1456884)
Supplement: Supplementary file 3 [file Table_3.docx]

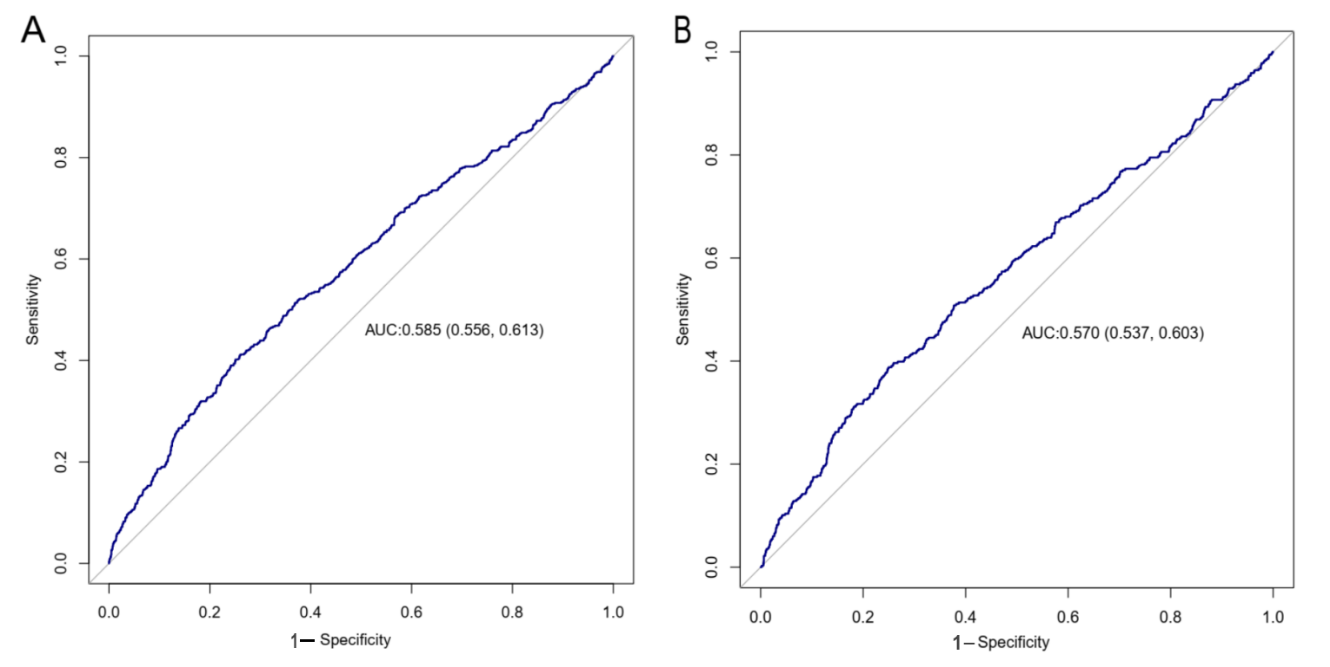


**Supplemental Figure 1** ROC curve analysis of PRR predicting all-cause mortality in patients with non-traumatic cerebral hemorrhage. (A) ROC for hospital mortality (B) ROC for ICU mortality. PRR, platelet-to-red cell distribution width ratio
